# F_1_F_0_-ATP Synthase Inhibitory Factor 1 in the Normal Pancreas and in Pancreatic Ductal Adenocarcinoma: Effects on Bioenergetics, Invasion and Proliferation

**DOI:** 10.3389/fphys.2018.00833

**Published:** 2018-07-11

**Authors:** Helen Tanton, Svetlana Voronina, Anthony Evans, Jane Armstrong, Robert Sutton, David N. Criddle, Lee Haynes, Michael C. Schmid, Fiona Campbell, Eithne Costello, Alexei V. Tepikin

**Affiliations:** ^1^Department of Cellular and Molecular Physiology, University of Liverpool, Liverpool, United Kingdom; ^2^Department of Molecular and Clinical Cancer Medicine, University of Liverpool, Liverpool, United Kingdom

**Keywords:** pancreatic ductal adenocarcinoma, pancreatic acinar cells, F_1_F_0_-ATP synthase inhibitory factor 1, bioenergetics, ATP, mitochondrial membrane potential

## Abstract

F_1_F_0_-ATP synthase inhibitory factor 1 (IF1) inhibits the reverse mode of F_1_F_0_-ATP synthase, and therefore protects cellular ATP content at the expense of accelerated loss of mitochondrial membrane potential (ΔΨm). There is considerable variability in IF1 expression and its influence on bioenergetics between different cell types. High levels of IF1 in a number of cancers have been linked to increased glycolysis, resistance to cell death, increased migration and proliferation. However, neither the expression nor role of IF1 in the normal pancreas or in pancreatic cancer has been characterized. In this study, we found that pancreatic ductal adenocarcinoma (PDAC) patients express higher levels of IF1 in cancerous cells than in pancreatic acinar cells (PACs). PDAC cell lines have a higher IF1 content and IF1/ATP synthase ratio than PACs. The observed differences are consistent with the ability of the respective cell types to maintain ΔΨm and ATP levels in conditions of chemical hypoxia. Acinar cells and PDAC cells preferentially express different IF1 isoforms. Both knockdown and knockout of IF1 in the PANC-1 pancreatic cancer cell line modified cellular bioenergetics and decreased migration, invasion and proliferation suggesting the putative importance of IF1 for PDAC growth and metastasis.

## Introduction

F_1_F_0_-ATP synthase inhibitory factor 1 is a small, heat stable protein capable of interacting with F_1_F_0_-ATP synthase (Pullman and Monroy, 1963; Cabezon et al., 2000b; Campanella et al., 2008). The F_1_F_0_-ATP synthase plays a major role in OXPHOS and is the main ATP producing enzyme in the majority of mammalian cells [reviewed in (Walker, 2013)]. ATP synthesis by F_1_F_0_-ATP synthase is coupled to H^+^ transfer via its F_0_ domain and requires a significant electrochemical gradient, formed by the respiratory complexes of the ETC (Walker, 2013). Under conditions in which mitochondrial respiration is compromised, such as during oxygen deprivation (e.g., as a result of ischemia), the electrochemical gradient collapses and it becomes energetically favorable for the F_1_F_0_-ATP synthase to work in reverse as an ATP-hydrolysing proton pump (Green and Grover, 2000; Vinogradov, 2000; Campanella et al., 2008). This reverse mode can sustain the Δψ_m_, but is coupled to undesirable hydrolysis of ATP (Campanella et al., 2008). In some cell types approximately 80–90% of ATP consumption during ischaemic conditions has been attributed to hydrolysis by the F_1_F_0_-ATP synthase (Rouslin et al., 1990; Di Pancrazio et al., 2004). Mitochondrial ATP consumption can lead to cell death, especially in metabolically active tissues that do not have a high glycolytic capacity (Jennings and Reimer, 1991; Grover et al., 2008).

Hydrolysis of ATP by F_1_F_0_-ATP synthase is inhibited by IF1 (Pullman and Monroy, 1963; Cabezon et al., 2000b) a highly conserved protein, encoded by the *ATPIF1* gene (Ichikawa et al., 1999; Martinez-Reyes and Cuezva, 2014). Variable splicing of the IF1 mRNA results in IF1 isoforms 1, 2 and 3 [reviewed in (Garcia-Bermudez and Cuezva, 2016)]. IF1 binds to the F_1_ domain of F_1_F_0_-ATP synthase with a 1:1 stoichiometry, and inhibits ATPase activity in a reversible and non-competitive manner (Green and Grover, 2000). Inhibition of F_1_F_0_-ATP synthase by IF1 is pH dependent; at a pH value of 6.5 or below, IF1 is present within mitochondria in its active dimeric state (Cabezon et al., 2000a). Optimal inhibition by IF1 is between pH 6.5 and 6.7, a level reached in the mitochondria during ischaemic conditions (Rouslin, 1983). At higher pH, IF1 dimers form tetramers, a structure which masks residues 14–47 – the inhibitory region of the protein – and therefore renders IF1 inactive (Cabezon et al., 2000a, 2001). IF1 has been shown to decrease ATP hydrolysis by the F_1_F_0_-ATP synthase by up to 80–90% (Rouslin et al., 1990; Garcia et al., 2006), and can therefore considerably protect cells from ischaemic injury and death. The level of IF1 expression naturally varies in tissues and cell types depending on how metabolically active they are, and therefore dictates their response to hypoxia (Campanella et al., 2008).

F_1_F_0_-ATP synthase inhibitory factor 1 expression is upregulated in a number of human cancers (Sanchez-Cenizo et al., 2010; Sanchez-Arago et al., 2013; Wu et al., 2015; Yin et al., 2015; Gao et al., 2016; Santacatterina et al., 2016). In cancer cells, increased IF1 expression is associated with metabolic reprogramming (Sanchez-Cenizo et al., 2010), resistance to apoptosis (Formentini et al., 2012; Faccenda et al., 2013; Santacatterina et al., 2016), increased invasion (Wu et al., 2015; Yin et al., 2015) and increased proliferation (Formentini et al., 2012; Sanchez-Arago et al., 2013; Yin et al., 2015; Santacatterina et al., 2016). In addition, previous studies have reported that high IF1 expression correlates with poor prognosis and reduced survival, demonstrating its potential use as a predictive marker (Sanchez-Arago et al., 2013; Song et al., 2014; Wu et al., 2015; Yin et al., 2015; Gao et al., 2016). It should be noted, however, that in a number of cancer types high IF1 was associated with increased patient survival (Sanchez-Arago et al., 2013) and that some IF1 effects are controversial (Fujikawa et al., 2012).

Pancreatic cancer is the 7th most common cause of cancer-related death globally (Ferlay et al., 2015) with PDAC accounting for the majority (∼85%) of cases. Understanding the cellular mechanisms of carcinogenesis is paramount for the development of treatment against this type of cancer. Changes of IF1 expression during malignant transformation of the exocrine pancreas and its effects on cellular bioenergetics, proliferation and invasion of PDAC cells have not yet been described. This therefore became the focus of our study.

## Materials and Methods

### Chemicals

Oligomycin was purchased from Cayman Chemical; Paraformaldehyde (16%) was obtained from Agar Scientific, and Propidium iodide from Thermo Fisher Scientific. Antimycin, CCCP, TMRM, Iodoacetate, Collagenase and Triton-x were all purchased from Sigma. All chemicals used were of analytical grade.

### Cell Culture

The human pancreatic cancer cell lines, PANC-1, MIA PaCa-2 and BxPC-3 (American Type Culture Collection, CRL-1469, CRL-1420 and CRL-1687 respectively), were cultured in complete Dulbecco’s modified Eagle medium (DMEM) supplemented with 10% (v/v) fetal bovine serum (FBS), 100 units/ml penicillin, 100 μg/ml streptomycin and 292 μg/ml glutamine (all from Thermo Fisher Scientific). Primary murine pancreatic cancer cells were isolated from tumors arising in the Kras; p53; Pdx-Cre mouse model (KPC) as previously described (Olive et al., 2009). KPC-derived PDAC cells were cultured in complete DMEM and used at a low passage (<10). HPDE cells were purchased from Kerafast (Boston, MA, United States). The specific HPDE cell line (H6c7, catalog number ECA001) was cultured in 1x Keratinocyte-SFM supplemented with human recombinant epidermal growth factor 1-53 (EGF-153) and Bovine pituitary extract (BPE) (Thermofisher Scientific). All cell lines were cultured at 37°C with 5% CO_2_ in a humidified incubator.

### Mouse Tissue and Primary Cells

Tissues and pancreatic acinar cells (PACs) were obtained from 6-week-old, male CD1 and C57BL6/J mice (Charles River). The animals were humanely sacrificed by cervical dislocation (schedule 1 procedure) in accordance with the Animals (Scientific Procedures) Act (1986) under Establishment License 40/2408 and with approval by the University of Liverpool Animal Welfare Committee and Ethical Review Body. For isolation of PACs, pancreata were digested using 200 unit/ml collagenase as previously described (Osipchuk et al., 1990).

### Immunofluorescence Studies

Immunofluorescence staining was performed on cells fixed with 4% Paraformaldehyde, permeabilised with 0.2% Triton-x and blocked with 10% Goat serum and 1% Bovine Serum Albumin in phosphate buffered saline (PBS). The primary antibodies used were IF1 [5E2D7] (ab110277, Abcam), IF1 (12067-1-AP, Proteintech) and ATPsβ (ab14730, Abcam). Goat anti-mouse (H + L) lgG (Alexa Fluor 488, A-11001 Invitrogen) and Goat anti-rabbit (H + L) lgG (Alexa Fluor 647, A-21244 Invitrogen) were used as secondary antibodies.

### Immunohistochemistry

Formalin-fixed and paraffin-embedded PDAC tissue sections (from 17 patients) were obtained from NIHR Liverpool Pancreas Biomedical Research Unit (PBRU). All patients gave written informed consent using approved ethics protocols, at the Royal Liverpool University Hospital. The study was approved by the National Research Ethnics Service Committee North West- Cheshire 11/NW/0083. All procedures were performed in accordance with the relevant guidelines and regulations. The samples were deparaffined and underwent antigen retrieval in a PT link (Dako). Samples were blocked with peroxidase block solution (Dako envision kit) and incubated with IF1 (mouse 12067-1-AP, Proteintech) and ATPsβ (rabbit ab14730, Abcam) antibodies. All samples were stained with the respective secondary antibodies (Dako envision kit) and counterstained with haematoxylin.

### Western Blot Analysis

A Pierce BCA protein assay kit (Thermo Fisher Scientific) was used to determine the protein concentration of lysed samples against Bovine serum albumin standards of known concentration. Samples containing equal amounts of protein were electrophoresed on SDS–PAGE gels, and transferred to nitrocellulose membrane. IF1 (ab110277, Abcam), ATPsβ (ab14730, Abcam), Calnexin (C4731, Sigma) and Vimentin (V6630, Sigma) primary antibodies, and peroxidase-conjugated anti-mouse (A5278, Sigma) and anti-rabbit (A6154, Sigma) secondary antibodies were used for the immunoblotting assays. Bands were visualized with enhanced chemiluminescence (ECL) Western blotting substrates (ThermoScientific) and a ChemiDoc XRS + molecular imaging system (Bio-Rad). The pixel intensities of the bands were calculated using ImageLab software.

### Mitochondrial Membrane Potential Measurements

A Zeiss 510 microscope (63× water immersion objective, NA = 1.4) was used to record the ΔΨm of pancreatic acinar, PANC-1, MIA PaCa-2 and BxPC3 cells using TMRM. TMRM has a delocalised positive charge and therefore accumulates in the mitochondria due to its negative membrane potential with respect to the cytosol. PDAC cell lines were seeded into 35 mm glass-bottom dishes (MatTek) the day before the experiment. PACs were isolated from the pancreas of a CD1 mouse on the day of the experiment, and 120 μl of cell suspension was seeded into glass-bottom dishes coated with Poly-L-lysine. The acinar cells were then left in the dishes for approximately 30–40 min, to allow attachment to the Poly-L-lysine coated glass surface.

Before experiments all cell types were incubated in the standard extracellular solution (see below) containing 40 nM TMRM for 30 min. Glass-bottom dishes containing cells were then placed onto the stage of an inverted confocal microscope and connected to a gravity-fed perfusion system in order to apply the specified extracellular solutions (see below). TMRM fluorescence was excited using a 543nm laser line, and emission was collected at wavelengths above 560 nm. Experiments were conducted at approximately 30°C. The confocal pinhole was set to 1.5 airy units.

The ETC inhibitor Antimycin (5 μM), the F_1_F_0_-ATP synthase inhibitor Oligomycin (5 μM), and the uncoupler CCCP (10 μM) that were used in these experiments were diluted into the standard extracellular solution, which was composed of 140 mM NaCl, 10 mM Hepes, 4.7 mM KCl, 1.3 mM MgCl_2_, 1 mM CaCl_2_, and 10 mM D-glucose. The solution was adjusted to pH 7.3 using NaOH. All extracellular solutions contained 40 nM of TMRM.

### ATP Measurements

For experiments with PDAC cell lines, 1ml of cell suspension containing 30,000 PDAC cells in DMEM was seeded into the individual wells of a 24-well plate the day before the experiment. For experiments with PACs, 1ml of cell suspension containing 75,000 freshly isolated acinar cells was seeded into individual wells of a 24-well plate 30–40 min before the experiment to allow cell attachment. Experiments were conducted at 35°C. At the time of the experiment the cells were incubated in the standard HEPES-based extracellular solution supplemented with the specified inhibitors or with the corresponding vehicle controls (0.05 or 0.1% ethanol). Following incubation, the extracellular solutions were removed and 100 μl of ATP-releasing buffer (FLSAR, Sigma) was added to each well. The plate was gently rocked back and forth for 30 s to aid ATP-release. The ATP assay kit (A22066, ThermoScientific) was used to measure the concentration of ATP according to the manufacturer’s instructions. This sensitive assay quantifies ATP by measuring the bioluminescence of a solution after addition of luciferin and luciferase. A POLARstar Omega plate reader (BMG Labtech) was used to record bioluminescence [for further details see (Armstrong et al., 2018)]. Changes in ATP were calculated relative to the respective vehicle controls. An inhibitor mixture of 5 μm Antimycin, 5 μm Oligomycin and 2mM Iodoacetate was used as a positive control for the assay; this mixture results in robust ATP depletion.

### Cell Transfection

PANC-1 and MIA PaCa-2 cells were transfected with adenoviral constructs (∼1 × 10^7^ PFU/ml and ∼3 × 10^7^ PFU/ml respectively) containing either IF1 short hairpin (sh)RNA or non-targeting (NT) shRNA (both from Vector Biolabs). After transfection, cells were cultured for 96 h, to achieve > 75% IF1 knockdown, before usage in any functional experiments.

### Knockout of IF1 in PANC-1 Cells

GenScript (NJ, United States) were recruited to create a permanent biallelic knockout of the ATPIF1 gene in the PANC-1 cell line using CRISPR/Cas9 genome editing. gRNA expression plasmids, designed to target the ATPIF1 loci, were generated and co-transfected into PANC-1 cells with cas9 expression plasmids. Knockout efficiency in transfected cells was determined by Sanger sequencing. Single cell clones with successful biallelic ATPIF1 knockout were generated. Knockout was confirmed using DNA sequence confirmation and Western blot analysis. GenScript also provided Wild-type (WT) PANC-1 cells that were used as the respective PANC-1 controls for all experiments with IF1-/- cells. Cell line authentication was carried out on the WT cells, IF1-/- cells, and the PANC-1 cells obtained from ATCC to verify that the identity of these cell lines were PANC-1.

### Migration and Invasion Assays

Transwell cell migration and invasion assays were performed in 24-well plates with 8 μm pore cell culture inserts (Corning). DMEM supplemented with 5% FBS was added to each well. For the migration assays, PANC-1 cells (3 × 10^4^ per insert) and MIA PaCa-2 cells (7.5 × 10^4^ per insert) were seeded in serum-free DMEM on top of the cell culture inserts and left to migrate over a period of 16 h at 37°C with 5% CO_2_ air in a humidified incubator. Cotton buds were used to wipe the top of each membrane. The migrated cells on the bottom of each membrane were fixed with 100% methanol, stained with propidium iodide, and imaged with a 10x dry objective on an AOBS confocal microscope. Seven representative images of each membrane were captured and Cell Profiler software was used to count the migrated cells. For the invasion assays, PANC-1 cells (4.5 × 10^4^ per insert) and MIA PaCa cells (1.2 × 10^5^ per insert) were seeded on top of 8 μm pore cell culture inserts pre-coated with matrigel and left to invade for a period of 24 h. The remaining procedure was the same as that for the migration assay.

### Proliferation Assays

Proliferation assays were performed in 35mm sterile cell culture dishes. 20,000 cells were suspended in DMEM supplemented with 10% FBS and seeded into two separate dishes. 4–5 h when the cells had settled and adhered to the bottom of the plates, 20 representative images were captured for each dish using a Zeiss 510 microscope and a 10× dry objective. The same process was carried out on the dishes of cells 2 days later, and percentage proliferation was calculated. A colorimetric assay using the Cell counting kit-8 (NBS biologicals) was also used as a second technique to measure proliferation, and was carried out in accordance with the manufacturer’s instructions. Cells were seeded into 96 well plates (5000 cells per well) and left to proliferate for 48 hrs at 37°C with 5% CO_2_ in a humidified incubator. Cells were subsequently incubated with Cell counting kit-8 solution for 1 h before absorbance was measured at 450 nm.

### Statistical Analysis

Results are expressed as Mean ± SEM. Significance was evaluated with a Student’s *t*-test or one-way ANOVA followed by Dunnet’s test and differences were considered significant when *P* < 0.05 (*P* < 0.05 is indicated by ^∗^ in the relevant figures).

## Results

### The Distribution of IF1 and F_1_F_0_-ATP Synthase in Human PDAC Sections

**FIGURE 1:**
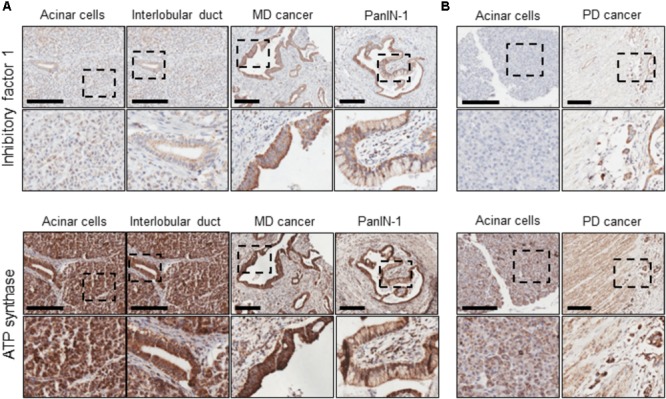
IF1 and ATP synthase distribution in normal pancreas and PDAC. Immunohistochemical staining for IF1 and ATPsβ. Scale bars: 200 μm. **(A)** Tissue sections from a patient with moderately differentiated (MD) PDAC. **(B)** Tissue sections from a patient with poorly differentiated (PD) PDAC. The figure demonstrates that IF1 expression is higher in moderately and poorly differentiated cancer and PanIN-1 than normal ducts and acinar cells. ATP synthase staining is present in all epithelial cell types. Note that for technical reasons, staining for IF1 and ATP synthase were conducted on neighboring tissue sections (approximately 5 μm apart). Negative controls for immunohistochemical staining are shown in Supplementary Figure 1.

The expression of IF1 and F_1_F_0_-ATP synthase in human PDAC tumor tissue sections was revealed by immunohistochemistry. Tissue sections from 17 patients were stained for IF1 (representative images are shown in **Figure 1**) and the intensity of antibody staining in different cell types was assessed by a specialist histopathologist. Normal PACs demonstrated weak cytoplasmic immunostaining with IF1 antibody compared to moderately differentiated tumor epithelium which demonstrated strong immunostaining (**Figure 1A**). IF1 immunostaining was seen in inter- and intra-lobular ducts but was also weaker than in cancerous tissue (**Figure 1A**). An example of PanIN-1 immunostaining is shown on the right panel of **Figure 1A**. Two foci of PanIN-1 were identified in the tissue sections, both revealing strong immunostaining. Poorly differentiated tumor epithelium also demonstrated a high intensity of IF1 immunostaining compared to normal acinar cells within the same tissue sections (**Figure 1B**). These results suggested that IF1 expression increases during pancreatic carcinogenesis and stimulated us to investigate IF1 expression and functions in normal and malignant pancreatic cells. Lower panels of **Figure 1** show immunostaining with an antibody against the ATPsβ. ATPsβ expression was present in all epithelial cell types (**Figure 1**).

### The IF1 and IF1/ATP Synthase Ratio in Pancreas, Heart and Liver

**FIGURE 2:**
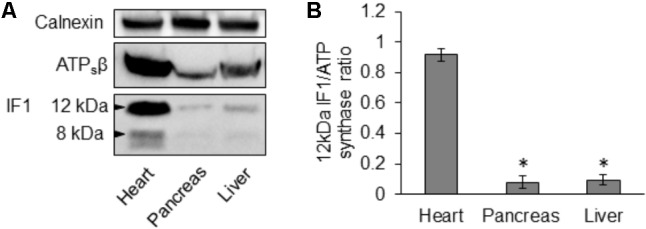
Expression of IF1 and ATP synthase in mouse tissues. **(A)** Western blot analysis shows expression of IF1 and ATPsβ in normal mouse heart, pancreas and liver tissue (representative of 3 biological repeats). Mouse heart tissue expresses the major 12- and 8-kDa protein isoforms of IF1; the 12-kDa isoform of IF1 is resolvable in pancreas and liver. Full-length blots are presented in Supplementary Figure 2. **(B)** The average IF1/ATPsβ ratios were calculated for each tissue using the major 12-kDa IF1 isoform; pancreas and liver tissue had a significantly lower IF1 and IF1/ATPsβ ratio than the heart (*n* = 3).

Using immunoblotting we compared IF1 and ATPsβ levels in lysates produced from mouse pancreas, heart and liver. We observed that the degree of IF1 staining and, importantly, the ratio of IF1 to its binding target, ATPsβ, differed substantially between these organs. In these experiments the heart was selected for comparison because cardiomyocytes have particularly strong expression of IF1 (Campanella et al., 2008). The 12- and 8-kDa isoforms of IF1 [(Sanchez-Arago et al., 2013; Garcia-Bermudez and Cuezva, 2016) see also GenBank: BC009677.1 and BC004955.1)] are clearly resolvable in heart tissue (**Figure 2A** and Supplementary Figure 2). Pancreas and liver tissue both demonstrated a significantly lower expression level of IF1 relative to ATPsβ than the heart tissue (**Figures 2A,B** and Supplementary Figure 2). Both 12- and 8-kDa isoforms of IF1 were significantly less prominent in pancreas and liver tissue (the liver allows comparison with another major gland), with the 8-kDa isoform barely detected.

### The IF1 and IF1/ATP Synthase Ratio in Pancreatic Acinar Cells and PDAC Cells

Immunofluorescence staining of the human pancreatic cancer cell line, PANC-1, revealed that IF1 co-localizes with ATPsβ with a Pearson coefficient of 92.0 ± 0.7% (*n* = 20) (Supplementary Figure 3). This confirmed that IF1 is localized to the mitochondria. With the intention of investigating the IF1/ATPsβ ratio in normal acinar and PDAC cells, immunofluorescence studies were then extended to include the human pancreatic cancer cell line BxPC3, murine KPC-derived cells, and primary PACs isolated from a CD1 mouse strain. The fluorescence intensities of IF1 and ATPsβ were measured and used to calculate the respective IF1/ATPsβ ratios in these cell types. This revealed that the three pancreatic cancer cell lines, PANC-1, BxPC3 and KPC-derived cells, had a significantly higher IF1/ATPsβ ratio than acinar cells isolated from healthy mouse pancreas (Supplementary Figure 4).

**FIGURE 3:**
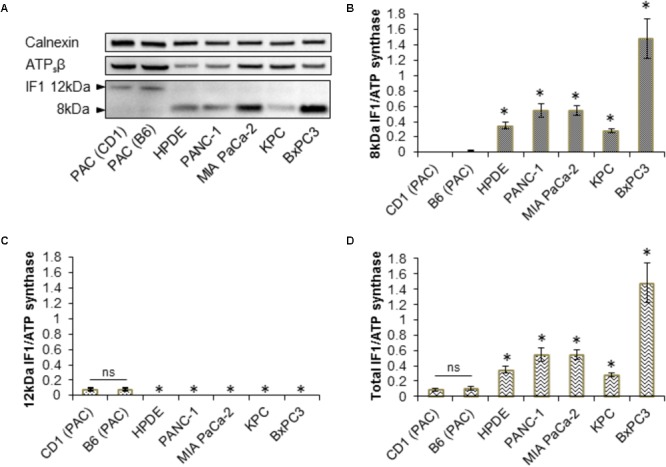
Expression of IF1 and ATP synthase in pancreatic acinar cells, and pancreatic cancer cell lines. **(A)** Western blot analysis of IF1 and ATPsβ expression. Pancreatic acinar cells (PAC) isolated from CD1 and C57BL/6 (B6) mice predominately express the 12-kDa protein isoform of IF1, whereas HPDE cells and the pancreatic cancer cell lines PANC-1, MIA PaCa-2, BxPC3, and KPC-derived cells, predominately express the 8-kDa protein isoform of IF1. Calnexin is shown as a loading control. Full-length blots are presented in Supplementary Figure 5. **(B)** Quantification of Western blot bands demonstrate that HPDE cells and the pancreatic cancer cell lines have significantly higher levels of the 8-kDa IF1 isoform and higher 8-kDa IF1/ATPsβ ratios than pancreatic acinar cells (PAC) (*n* = 5). **(C)** Quantification of Western blot bands demonstrate that HPDE cells and the pancreatic cancer cell lines have significantly lower levels of the 12-kDa IF1 isoform and lower 12-kDa IF1/ATPsβ ratios than pancreatic acinar cells (PAC) (*n* = 5). **(D)** Combining the sum of the 12- and 8-kDa IF1 isoform band intensities demonstrates that the HPDE cells and pancreatic cancer cell lines have significantly higher total IF1/ ATPsβ ratios than pancreatic acinar cells (PAC) (*n* = 5).

Western blot analysis for IF1 and ATPsβ was carried out on primary PACs isolated from CD1 and a C57BL6/J (B6) mice, the human pancreatic ductal epithelial cell line (HPDE), the human PDAC cell lines PANC-1, BxPC3, MIA PaCa-2 and on KPC-derived cells (**Figure 3A** and Supplementary Figure 5). These Western blots revealed that PACs isolated from a CD1 and B6 mouse predominately express the 12-kDa isoform of IF1, in contrast to the PDAC cell lines which almost exclusively express the 8-kDa isoform of IF1 (**Figures 3A–C** and Supplementary Figure 5). Ratios of the line intensities for individual isoforms of IF1 to that of ATPsβ are shown on **Figures 3B,C**. The total IF1/ATPsβ ratio, which includes the sum of line intensities for both IF1 isoforms, was significantly higher in HPDE cells and the pancreatic cancer cell lines than in PACs (**Figure 3D**). This is consistent with the outcomes of immunostaining experiments (see **Figure 1**).

Immediate early gene X-1 was reported to be involved in the degradation of IF1 (Shen et al., 2009). In our study we also observed a negative correlation between the IF1/ATPsβ ratio and IEX-1 expression in the cell types tested. PACs isolated from CD1 and B6 mice, which we have shown to have a low IF1/ATPs ratio, demonstrated robust IEX-1 expression. The PDAC cell lines PANC-1, BxPC3 and KPC-derived cells which have relatively high IF1/ATPsβ ratios, demonstrated little to no IEX-1 expression (Supplementary Figure 6).

### The IF1/ATP Synthase Ratio Determines the Response of ΔΨm to Mitochondrial Inhibitors

**FIGURE 4:**
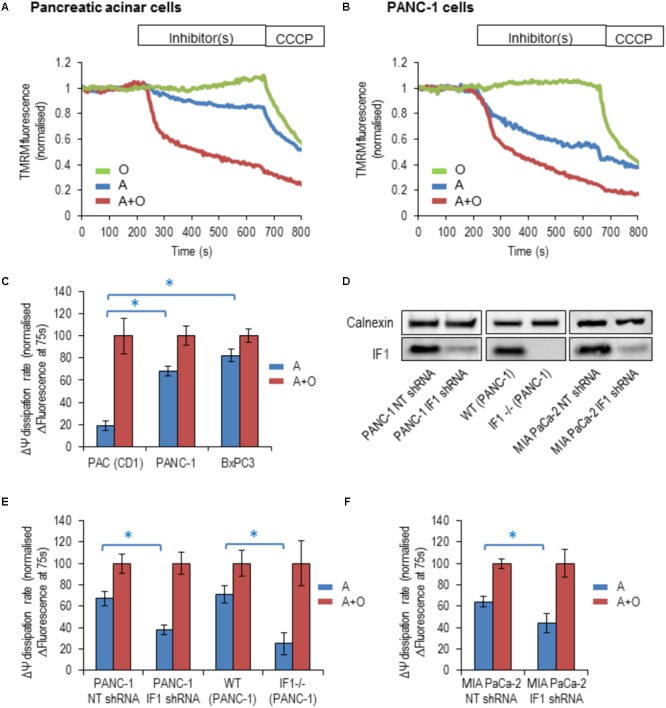
The response of the ΔΨm to the addition of mitochondrial inhibitors in pancreatic acinar cells and pancreatic cancer cells. Oligomycin (O; green), Antimycin (A; blue), or Antimycin + Oligomycin (A + O; red) and CCCP were utilized in these experiments. **(A)** Example traces of normalized TMRM fluorescence recorded from pancreatic acinar cells (PAC) isolated from CD1 mice. **(B)** Example traces of normalized TMRM fluorescence recorded from PANC-1 cells. **(C)** The bar graph summarizes the differences between the baseline TMRM fluorescence of the cells (PAC, PANC-1 and BxPC3), and their TMRM fluorescence recorded 75 s after the addition of A or A + O. This difference reflects the rate of ΔΨm dissipation (for PAC cells *n* = 27 for A and *n* = 12 for A + O; for PANC-1 cells *n* = 32 for A and *n* = 21 for A + O; for BxPC3 cells *n* = 39 for A and *n* = 48 for A + O). **(D)** Western blot analysis of IF1 (8-kDa) in PANC-1 and MIA PaCa-2 cells. The left panel illustrates efficiency of shRNA-mediated knockdown, whilst the middle panel demonstrates the complete IF1 knockout (by CRISPR/Cas9 gene editing) in PANC-1 cells. The right panel demonstrates efficiency of shRNA-mediated knockdown in MIA PaCa-2 cells. Full-length blots are presented in Supplementary Figure 7. **(E)** The bar graph summarizes the differences between the baseline TMRM fluorescence of the cells and their TMRM fluorescence recorded 75 s after the addition of A or A + O. This panel illustrates the difference in the rate of ΔΨm dissipation in PANC-1 cells following transfection with non-targeting (NT) shRNA (*n* = 32 for A and *n* = 21 for A + O) or IF1 shRNA (*n* = 25 for A and *n* = 20 for A + O). Similar changes were obtained for wild-type (WT) PANC-1 cells (*n* = 17 for A and *n* = 26 for A + O) and IF1-/- PANC-1 cells (*n* = 8 for A and *n* = 10 for A + O). **(F)** The bar graph summarizes the differences between the baseline TMRM fluorescence of the cells and their TMRM fluorescence recorded 75 s after the addition of A or A + O. This panel illustrates the difference in the rate of ΔΨm dissipation in MIA PaCa-2 cells following transfection with NT shRNA (*n* = 8 for A and *n* = 18 for A + O) or IF1 shRNA (*n* = 16 for A and *n* = 17 for A + O).

By inhibiting the reverse mode of F_1_F_0_-ATP synthase, IF1 is expected to accelerate the dissipation of the ΔΨm when the ETC is inhibited (Campanella et al., 2008). To reveal changes of ΔΨm, cells were incubated with TMRM (fluorescent probe with delocalised positive charge) (Voronina et al., 2004; Campanella et al., 2008). The ETC inhibitor, antimycin, the F_1_F_0_-ATP synthase inhibitor, oligomycin or a combination of both inhibitors were used in these experiments. The ETC uncoupler CCCP was added to the cells at the end of the experiment to completely collapse ΔΨm (**Figures 4A,B**). In PACs, the rate of loss of ΔΨm was substantially larger with the addition of antimycin and oligomycin in combination, than with the addition of antimycin alone (**Figures 4A,C**). The difference in the rates was much smaller in BxPC3 and PANC-1 cells, which is consistent with the high levels of IF1 in the mitochondria of these cell types (**Figures 4B,C**).

We next tested the effect of reduction in IF1 expression on ΔΨm responses. Immunoblotting was used to confirm that IF1 shRNA down-regulated the expression of IF1 in PANC-1 cells (by 75–90%) compared to control cells transfected with NT shRNA (**Figure 4D** left panel and Supplementary Figure 7A). In these experiments the ΔΨm changes in PANC-1 cells that were transfected with NT shRNA (PANC-1 NT shRNA) or with IF1 shRNA (PANC-1 IF1 shRNA) were compared. The ΔΨm changes in these cell types were recorded after application of the same inhibitors. PANC-1 NT shRNA cells demonstrated very similar responses to the inhibitors as PANC-1 cells (compare **Figures 4C,E**). However, PANC-1 IF1 shRNA cells demonstrated a larger difference in their normalized fluorescence changes when antimycin and oligomycin were added in combination, than when antimycin was added alone (**Figure 4E**).

Using IF1 knockout in PANC-1 cells we further investigated the putative effect of IF1 on ΔΨm. Immunoblotting was also used to confirm that IF1-/- PANC-1 cells did not demonstrate any IF1 expression (see **Figure 4D** middle panel and Supplementary Figure 7B). We compared ΔΨm responses to mitochondrial inhibitors of WT PANC-1 cells with those of IF1-/- PANC-1 cells (**Figure 4E**). We observed a significantly larger difference between ΔΨm responses to antimycin and the combination of antimycin + oligomycin in IF1-/- PANC-1 than in WT PANC-1 (**Figure 4E**). To investigate the effect of IF1 expression on the ΔΨm in an additional PDAC cell line, we compared the ΔΨm responses of MIA PaCa-2 cells transfected with NT shRNA (MIA PaCa-2 NT shRNA) with those transfected with IF1 shRNA (MIA PaCa-2 IF1 shRNA). Immunoblotting was used to confirm that IF1 shRNA down-regulated the expression of IF1 in MIA PaCa-2 cells (see **Figure 4D** right panel and Supplementary Figure 7C). Similar to the results seen in PANC-1 cells, we observed a significantly larger difference between the ΔΨm responses to antimycin and a combination of antimycin + oligomycin in IF1 shRNA MIA PaCa-2 cells than in NT shRNA MIA PaCa-2 cells (**Figure 4F**).

The findings of these experiments are consistent with the reported inhibitory action of IF1 on the reverse mode of F_1_F_0_- ATP synthase (Campanella et al., 2008).

### IF1 Levels and ATP Content in Cells Subjected to Mitochondrial Inhibitors

**FIGURE 5:**
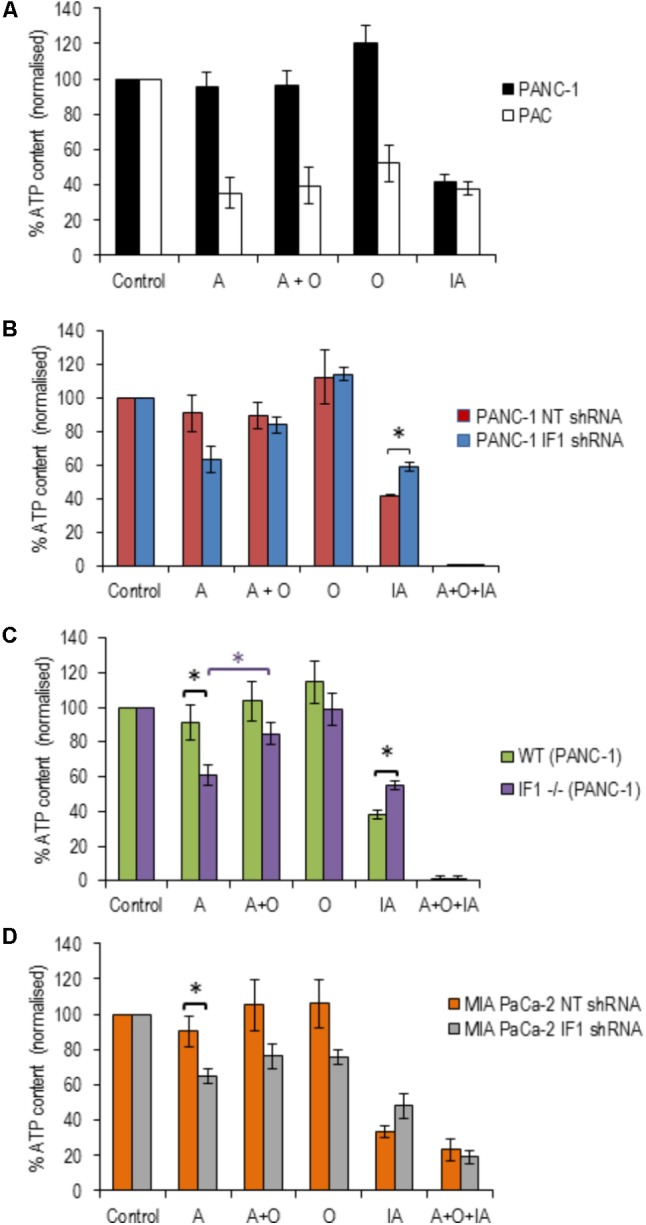
The effect of inhibitors of ATP production on ATP levels in pancreatic acinar cells and PDAC cells (with normal, reduced or eliminated IF1). ATP levels in the respective vehicle controls (control measurements) were assumed to be 100% (left bars in **A**–**D**) and all measurements in individual cell types were normalized to their corresponding controls **(A–D)**. **(A)** To reveal the effect of ETC, F_1_F_0_-ATP synthase and glycolysis inhibitors on ATP levels, PANC-1 and pancreatic acinar cells (PAC) were incubated with Antimycin (A), Oligomycin (O), Antimycin + Oligomycin (A + O), or Iodoacetate (IA) for 20 min before ATP was extracted and measured (*n* = 6 for both cell types). **(B)** PANC-1 cells transfected with NT or IF1 shRNA (*n* = 6 for both conditions) were incubated with A, O, A + O, IA or A + O + IA for 20 min before ATP was extracted and measured. **(C)** Wild-type (WT) and IF1 -/- PANC-1 cells were incubated with A, O, A + O, IA or A + O + IA for 20 min before ATP was extracted and measured. **(D)** MIA PaCa-2 cells transfected with NT or IF1 shRNA (*n* = 4 for both conditions) were incubated with A, O, A + O, IA, or A + O + IA for 20 min before ATP was extracted and measured.

Pancreatic acinar cells and PANC-1 cells were subjected to 20-min incubation with the following inhibitors; antimycin, oligomycin, a combination of antimycin + oligomycin, and iodoacetate (an inhibitor of glyceraldehyde-3-P-dehydrogenase). PACs demonstrated large decreases (50–70%) in their ATP levels in response to all inhibitors tested (**Figure 5A**), whilst PANC-1 cells only demonstrated a decrease in ATP levels in response to iodoacetate (**Figure 5A**). This is compatible with the notion that PACs have substantial oxidative and glycolytic components of ATP production (Voronina et al., 2010), whilst PDAC cells are largely glycolytic (James et al., 2015).

We next tested the effect of IF1 knockdown on the bioenergetics of PANC-1 cells. PANC-1 cells transfected with either NT or IF1 shRNA were also subjected to 20-min incubation periods with antimycin, oligomycin, a combination of antimycin + oligomycin or iodoacetate. In these experiments we also used one extra inhibitor mixture which contained antimycin, oligomycin and iodoacetate; this was applied as a positive control for ATP depletion since both OXPHOS and glycolysis are inhibited by the compounds present in this mixture. The responses of PANC-1 cells transfected with NT shRNA were very similar to those seen in PANC-1 cells that had not undergone transfection. The responses of PANC-1 cells transfected with IF1 shRNA were not drastically different to those of PANC-1 cells and PANC-1 cells transfected with NT shRNA (**Figure 5B**). Antimycin caused a decrease in ATP levels in IF1 shRNA cells compared to that of NT shRNA cells, (**Figure 5B**) but the difference was not statistically significant (*p* = 0.07). There was also no statistically significant difference between ATP levels following antimycin and antimycin + oligomycin treatment of IF1 shRNA cells (**Figure 5B**).

We were able to resolve the effect of IF1 knockout on the bioenergetics of PANC-1 cells. Antimycin significantly reduced ATP content in IF1-/- cells in comparison with the WT cells (**Figure 5C**) and there was a statistically significant difference in ATP content of IF1-/- cells between the antimycin and antimycin + oligomycin treatments. We next tested the effect of IF1 knockdown on the bioenergetics of another PDAC cell line MIA PaCa-2 and found modest (but statistically significant) potentiation of the effect of antimycin (**Figure 5D**, compare NT shRNA and IF1 shRNA cells). We did not however find statistically significant difference in ATP content between antimycin and antimycin + oligomycin treatments in MIA PaCa-2 cells (**Figure 5D**). Both knockout and knockdown of IF1 in PANC-1 cells potentiated the effect of iodoacetate on ATP levels (**Figures 5B,C**). Knockdown of IF1 in MIA PaCa-2 cells also reduced the mean ATP content observed in the presence of iodoacetate but the effect was not statistically significant (**Figure 5D**). Overall, these results suggest that IF1 can modulate ATP changes in PDAC cells, although the effects of IF1 were less prominent than responses reported for some primary cell types (e.g., Rouslin et al., 1990; Campanella et al., 2008).

### Reduction or Removal of IF1 Decreases Migration, Invasion and Proliferation of PANC1 Cells

**FIGURE 6:**
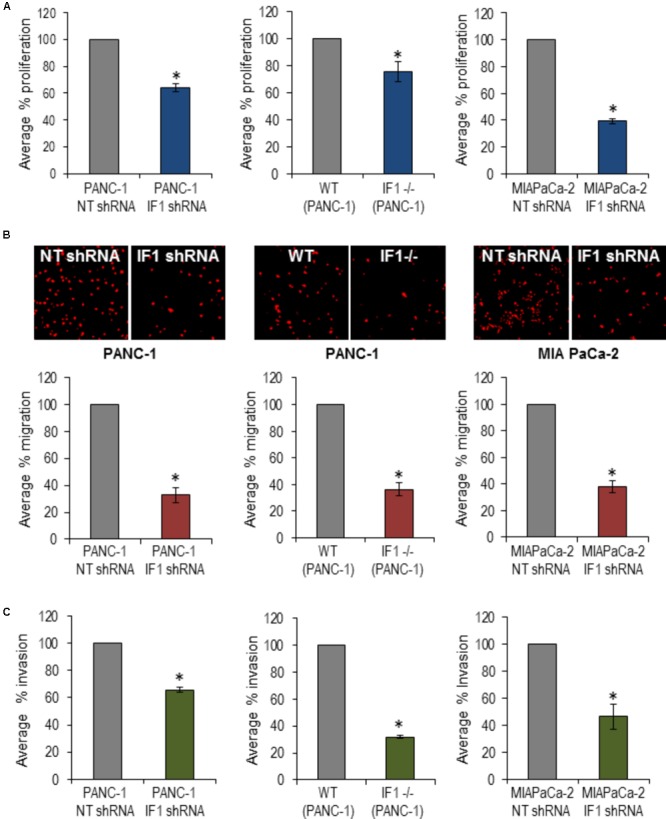
The effect of IF1 knockdown and knockout on proliferation, migration and invasion of pancreatic cancer cell lines. **(A)** Proliferation, determined using a Cell counting kit-8 based assay. The percentage proliferation of PANC-1 cells transfected with IF1 shRNA was normalized to that of cells transfected with NT shRNA, for each individual experiment (*n* = 3). The percentage proliferation of IF1-/- PANC-1 cells was normalized to that of WT PANC-1 cells for each individual experiment (*n* = 3). The percentage proliferation of MIA PaCa-2 cells transfected with IF1 shRNA was normalized to that of cells transfected with NT shRNA for each individual experiment (*n* = 3). **(B)** Cell migration, determined by the number of cells migrated through the porous membrane of Boyden chambers. The percentage migration of PANC-1 cells transfected with IF1 shRNA was normalized to that of cells transfected with NT shRNA cells for each individual experiment (*n* = 4). The percentage migration of IF1-/-PANC-1 cells was normalized to that of cells WT PANC-1 cells for each individual experiment (*n* = 4). The percentage migration of MIA PaCa-2 cells transfected with IF1 shRNA was normalized to that of cells transfected with NT shRNA cells for each individual experiment (*n* = 3). Representative images of propidium iodide stained cells on the outer surface of the Boyden chamber membrane are shown for each cell type. **(C)** Cell invasion, determined by the number of cells that invaded through the porous membrane of matrigel-coated Boyden chamber. The percentage invasion of PANC-1 cells transfected with IF1 shRNA was normalized to that of cells transfected with NT shRNA cells for each individual experiment (*n* = 5). The percentage invasion of IF1-/-PANC-1 cells was normalized to that of cells WT PANC-1 cells for each individual experiment (*n* = 4). The percentage invasion of MIA PaCa-2 cells transfected with IF1 shRNA was normalized to that of cells transfected with NT shRNA cells for each individual experiment (*n* = 3).

To investigate a putative functional role of IF1 in pancreatic cancer, PANC-1 cells were transfected with NT shRNA or IF1 shRNA and subsequently used in migration, invasion and proliferation assays. WT PANC-1 cells and IF1-/- PANC-1 cells were also used in these assays, as well as MIA PaCa-2 cells transfected with NT shRNA or IF1 shRNA. Functional assays demonstrated that IF1 knockdown significantly reduced proliferation (**Figure 6A** and Supplementary Figure 8), migration (**Figure 6B**), and invasion (**Figure 6C**) of PANC-1 cells and MIA PaCa-2 cells compared to the corresponding NT shRNA transfected control cells (**Figures 6A–C**). Similar decreases of migration, invasion and proliferation were observed in IF1-/- PANC-1 cells compared to WT controls (**Figures 6A–C** and Supplementary Figure 8). Prominent changes in migration, invasion and proliferation of PANC1 cells, attained as a result of reduction or removal of IF1 suggest the importance of this protein for growth and metastasis of PDAC tumors.

## Discussion

Some normal human tissues and cell types such as the endometrium and kidney have a naturally high expression of IF1 (Sanchez-Arago et al., 2013). Neurons have a higher IF1/ATP synthase ratio compared to astrocytes (Campanella et al., 2008). Heart is another tissue type with high IF1 expression and a high IF1/ ATP synthase ratio (Campanella et al., 2008) and was used as a positive control in our study. We found that the pancreas, of which more than 80% is composed of acinar cells (Bolender, 1974), has relatively low IF1 and IF1/ATP synthase ratio in comparison with the heart. The level of IF1 expression appears to be dependent on the metabolic demand and glycolytic potential of the particular tissue or cell type. Normal tissues and cells that are highly metabolically active and do not have a large glycolytic capacity, tend to have higher IF1/ATP synthase ratios than those that have a lower metabolic demand and/or the ability to efficiently use glycolysis for their ATP production (Campanella et al., 2009). Pancreatic acinar cells have significant glycolytic capacity (Voronina et al., 2010) therefore they can probably afford to have relatively low IF1 levels, as observed in our study.

Increased IF1 expression has been observed in a number of cancers compared to corresponding normal cells and tissues (Sanchez-Cenizo et al., 2010; Formentini et al., 2012; Sanchez-Arago et al., 2013; Song et al., 2014; Wu et al., 2015; Yin et al., 2015; Gao et al., 2016; Santacatterina et al., 2016). This increase in IF1 expression accompanies and probably facilitates malignant transformation (Sanchez-Cenizo et al., 2010; Formentini et al., 2012; Song et al., 2014; Santacatterina et al., 2016). Our results certainly indicate that PDAC belongs to this group of IF1-enriched cancers. One of the mechanisms that can determine the expression level of IF1 is IEX-1; this protein is considered to be responsible for targeting IF1 for degradation by a mitochondrial protease (Shen et al., 2009). In our study, strong IEX-1 expression was observed in pancreatic acinar cells whilst IEX-1 levels were low in all types of PDAC cells, demonstrating that IEX-1 negatively correlated with IF1 expression. These findings are consistent with the notion that IEX-1 is a regulator of IF1 levels. The effects of IEX-1 and other putative mechanisms of IF1 regulation in pancreatic cells are beyond the remit of the current study but will be further investigated in our laboratory.

**FIGURE 7:**
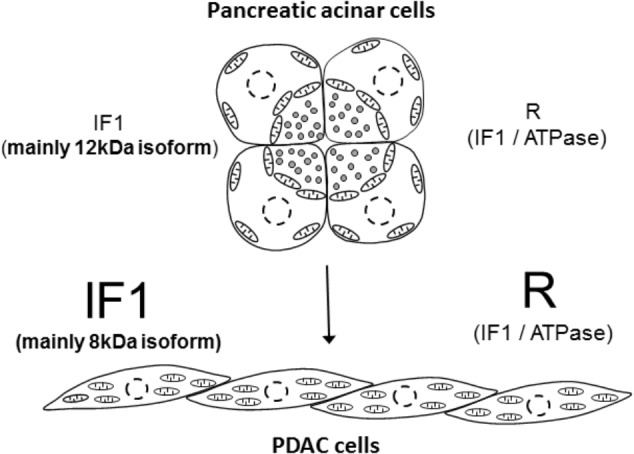
Summary figure. The figure illustrates the main finding of the study that pancreatic acinar cells contain low IF1 levels and have a low IF1 to ATP synthase ratio (symbol ‘R’ indicates ratio), whilst PDAC cells, which probably originate as a result of malignant transformation of pancreatic acinar cells, contain high levels of IF1 and have a high ratio of IF1 to ATP synthase. There is also a notable change in the expression of IF1 isoforms; pancreatic acinar cells preferentially express the 12-kDa isoform, whilst PDAC cells preferentially express the 8-kDa isoform. The putative process of transformation of the acinar cells to PDAC cells is indicated by the arrow.

Although controversial, there is reasonable evidence indicating that PDAC develops as a result of malignant transformation of pancreatic acinar cells [reviewed in (Rooman and Real, 2012)]. Results of our study indicate that this process is accompanied by an increase in IF1 content, an increase of the IF1/ATPsβ ratio and interestingly, by the shift of the main IF1 isoform; the 12-kDa isoform is preferentially expressed in normal cells whilst the 8-kDa isoform is preferentially expressed in PDAC cells (summarized in **Figure 7**). This prominent isoform shift seems to be specific for pancreatic carcinogenesis. It is important to note that increased IF1 expression in our study was also observed in HPDE cells, which is considered to be a nontumorigenic cellular model of ductal epithelium (Ouyang et al., 2000). This could indicate that an increase in IF1 levels accompanies early stages of acinar-to-ductal metaplasia (the process that is likely to be important for pancreatic cancer development (Rooman and Real, 2012; Storz, 2017). The high levels of IF1 observed in PanINs are consistent with this notion. The role of IF1 in acinar-to-ductal metaplasia and functions of IF1 in pancreatic ducts, which are known to be susceptible to mitochondrial damage in conditions of acute pancreatitis [(Biczo et al., 2011; Maleth et al., 2011) reviewed in (Maleth and Hegyi, 2016)], are interesting subjects for further investigations.

Due to its effect on bioenergetics, increased IF1 expression can increase the resilience of cancer cells, particularly in conditions of hypoxia (Campanella et al., 2008; Sanchez-Cenizo et al., 2010; Formentini et al., 2012). This is likely to be relevant to PDAC, which is poorly vascularised (Koong et al., 2000; Duffy et al., 2003; Kamphorst et al., 2015). The contribution of IF1 to bioenergetics can be revealed by inhibiting the ETC in the presence and absence of oligomycin. Oligomycin is a pharmacological inhibitor of both the forward and reverse mode of F_1_F_0_-ATP synthase, but in mitochondria with an inhibited ETC, oligomycin can only suppress the reverse mode. The levels of IF1 expression in different cell types appears to dictate the degree of ΔΨm preservation and ATP consumption when the ETC is disrupted (Campanella et al., 2008). This is consistent with our finding that the rate of ΔΨm dissipation is slower in cells with lower IF1 levels (PACs) than in cells with a higher IF1 content (BxPC3 and PANC-1). Similarity of the rates of ΔΨm dissipation in the presence and absence of F_1_F_0_-ATP synthase inhibitor (oligomycin) suggests efficient IF1-mediated inhibition of the reverse mode of F_1_F_0_-ATP synthase in PDAC cells. Furthermore, in PANC-1 cells, both knockdown (using specific shRNA) and knockout (using CRISPR/Cas9 gene editing) of IF1 significantly increased the difference between the rates of ΔΨm dissipation in the presence and absence of F_1_F_0_-ATP synthase inhibitor. Similar results were obtained with knockdown of IF1 in MIA PaCa-2 cells. These experiments further confirm the functional role of IF1.

The effects of IF1 knockdown in PANC-1 cells on the cytosolic ATP content were less prominent than the effect on ΔΨm dissipation. A possible interpretation of these results is that the remaining 20–25% of IF1 expression in IF1-shRNA treated PDAC cells is able to sufficiently reduce ATP hydrolysis by the reverse mode of F_1_F_0_-ATP synthase, making them difficult to distinguish from the control cells. An alternative interpretation is that the cells may accelerate their glycolytic ATP production to compensate for the ATP consumption by F_1_F_0_-ATP synthase. This interpretation is consistent with findings reported by Fujikawa and colleagues (Fujikawa et al., 2012) in which HeLa cells with knocked down IF1 expression demonstrated a relatively small and transient reduction in ATP. Importantly, knockout of IF1 in PANC-1 cells resulted in a statistically significant reduction of ATP content in response to antimycin. Furthermore, the addition of oligomycin (in combination with antimycin) partially reversed this effect. Knockdown of IF1 in MIA PaCa-2 cells resulted in modest but statistically significant reduction in ATP content following antimycin treatment (in comparison with control NT shRNA treated cells). Taken together, these results indicate that IF1 contributes to the bioenergetics and mitochondrial metabolism of PDAC cells, particularly when the ETC is inhibited (which can occur in conditions of hypoxia).

In addition to its effect on cellular bioenergetics, IF1 was reported to modify the proliferation (Formentini et al., 2012; Sanchez-Arago et al., 2013; Yin et al., 2015; Santacatterina et al., 2016), migration and invasion (Formentini et al., 2012; Song et al., 2014; Wu et al., 2015; Yin et al., 2015) of cancer cells. In our experiments, both knockdown and knockout of IF1 in PANC-1 cells, and knockdown of IF1 in MIA PaCa-2 cells, produced a statistically significant reduction in the proliferation of PANC-1 cells. Knockdown and knockout of IF1 both demonstrated prominent reductions in the migration and invasion of PANC-1 cells. Knockdown of IF1 also reduced migration and invasion of MIA PaCa-2 cells. Previous studies have linked IF1-mediated changes in migration and invasion, with a shift between the mesenchymal and epithelial phenotype of cancer cells (Song et al., 2014; Wu et al., 2015). In our study we were not able to resolve differences in the expression of mesenchymal marker (Vimentin) between PANC-1 cells and cells with reduced or eliminated IF1 (not shown, *n* = 3 for both knockdown and knockout). The relationship between IF1 and the migration/invasion of PDAC cells is therefore still enigmatic and constitutes the subject of further investigations in our laboratory.

This study reports low IF1 levels in normal acinar cells and high IF1 levels in pancreatic cancer cells suggesting that its expression increases during pancreatic carcinogenesis. Our results also indicate the importance of IF1 for bioenergetics, proliferation, migration and invasion of PDAC cells, suggesting its relevance for the growth and dissemination of PDAC.

## Author Contributions

HT, SV, AE, JA, MS, and FC contributed to the experimental part of the project. HT, RS, DC, LH, MS, FC, EC, and AT designed and developed the project.

## Conflict of Interest Statement

The authors declare that the research was conducted in the absence of any commercial or financial relationships that could be construed as a potential conflict of interest. The reviewer JM and handling Editor declared their shared affiliation.

## Abbreviations

ΔΨm: mitochondrial membrane potential
ATPsβ: β subunit of F1F0-ATP synthase
CCCP: carbonyl cyanide m-chlorophenyl hydrazone
ETC: electron transport chain
HPDE cells: human pancreatic ductal epithelial cells
IEX-1: immediate early gene X-1
IF1: F_1_F_0_-ATP synthase inhibitory factor 1 also known as ATP synthase inhibitory factor 1
OXPHOS: oxidative phosphorylation
PDAC: pancreatic ductal adenocarcinoma
TMRM: tetramethyl rhodamine methyl ester
