# Supplementary material for: F1F0-ATP Synthase Inhibitory Factor 1 in the Normal Pancreas and in Pancreatic Ductal Adenocarcinoma: Effects on Bioenergetics, Invasion and Proliferation
Source: Front Physiol. 2018 Jul 11;9:833. doi: 10.3389/fphys.2018.00833 (PMC6050379; doi:10.3389/fphys.2018.00833)
Supplement: Supplementary file 1 [file Presentation_1.PDF]

F_1F_0-ATP synthase Inhibitory Factor 1 in the normal pancreas and in pancreatic ductal adenocarcinoma: effects on bioenergetics, invasion and proliferation

Helen Tanton[1], Svetlana Voronina[1], Anthony Evans[2], Jane Armstrong[2], Robert Sutton[2], David N. Criddle[1], Lee Haynes[1], Michael C. Schmid[2], Fiona Campbell[2], Eithne Costello[2] and Alexei V.Tepikin[1*].

[1]Department of Cellular and Molecular Physiology and [2]Department of Molecular and Clinical Cancer Medicine, the University of Liverpool, Crown Street, Liverpool L69 3BX, UK.

SUPPLEMENTARY FIGURES

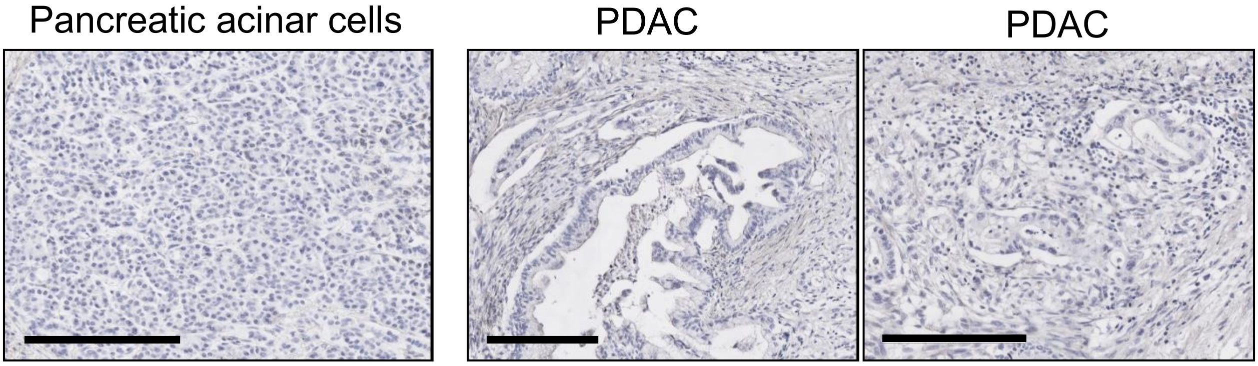

Supplementary Figure 1. Negative control for immunohistochemical staining.
The sections were treated in the same way as described in the main part of the manuscript but the step involving incubation with primary antibodies was omitted. The figure shows representative images of pancreatic acinar cells and PDAC tissue samples (n=4). Scale bars correspond to 200µm.

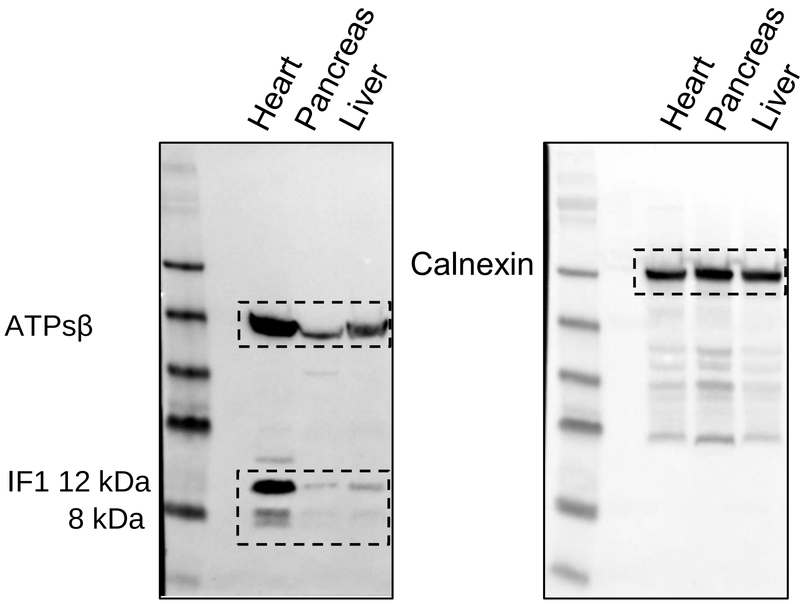

Supplementary Figure 2. Full-length blots of ATP synthase and IF1 in mouse tissues. Full-length blot for ATPsβ and IF1 corresponding to the Figure 2A in the main text; tissues lysates were firstly blotted for ATPsβ and IF1 (left panel), and then re-blotted for calnexin (right panel) as a loading control.

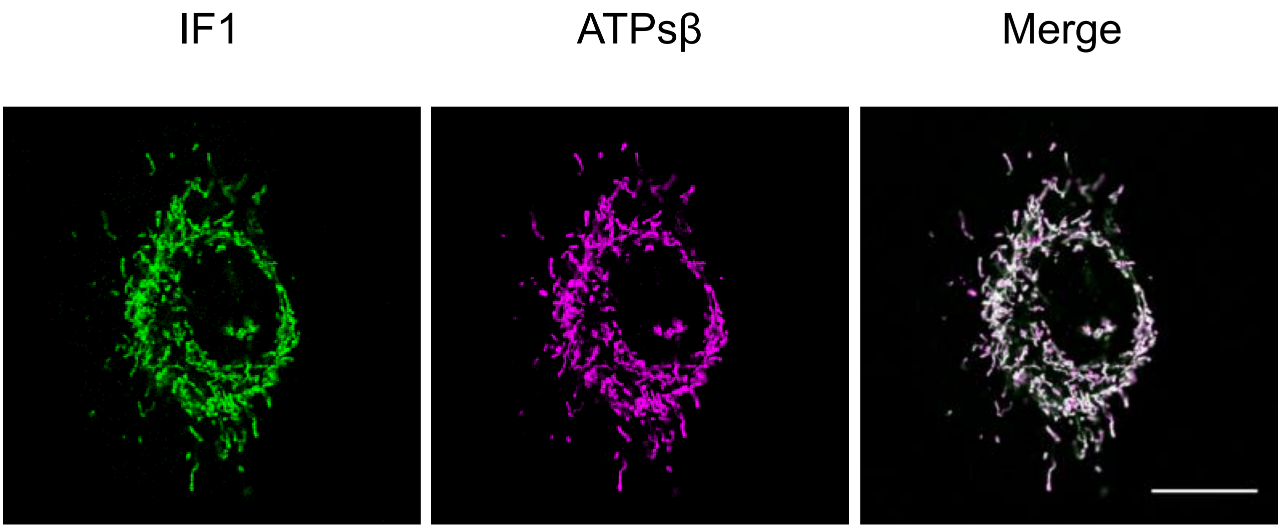

Supplementary Figure 3. Co-localisation of IF1 and ATP synthase in PANC-1 cells. Antibodies against IF1 (mouse 12067-1-AP, Proteintech) (green) and ATPsβ (rabbit ab14730, Abcam) (Magenta) co-localise in PANC-1 cells (Merge). Goat anti-mouse (H+L) lgG (Alexa Fluor 488, A-11001 Invitrogen) and Goat anti-rabbit (H+L) lgG (Alexa Fluor 647, A-21244 Invitrogen) were used as secondary antibodies. Scale bar: 20μm.

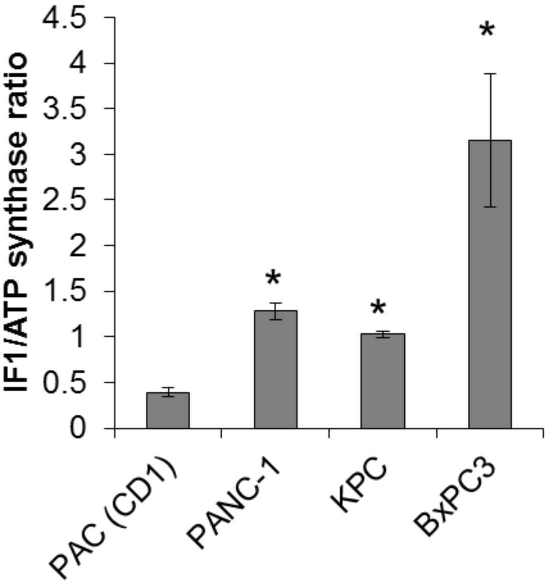

Supplementary Figure 4. IF1 and ATP synthase in pancreatic acinar cells and pancreatic cancer cell.
Quantification of the intensities of immunofluorescence staining. The average IF1/ATPsβ ratio of each pancreatic cancer cell line is significantly higher than that of pancreatic acinar cells (n=131 for PAC cells; n=112 for PANC-1 cells; n=221 for KPC cells; n= 171 for BxPC3 cells).

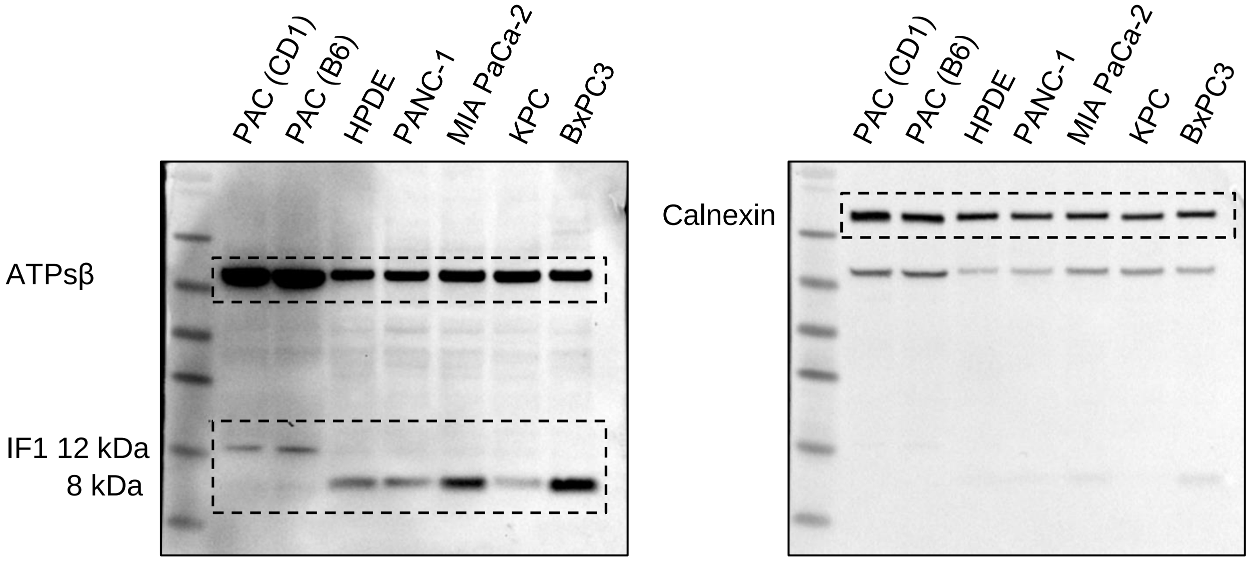

Supplementary Figure 5. Full-length blots of ATP synthase and IF1 in pancreatic acinar cells and pancreatic cancer cell lines. Full-length blot for ATPsβ and IF1 corresponding to the Figure 4A in the main text; cell lysates were firstly blotted for ATPsβ and IF1 (left panel), and then re-blotted for calnexin (right panel) as a loading control.

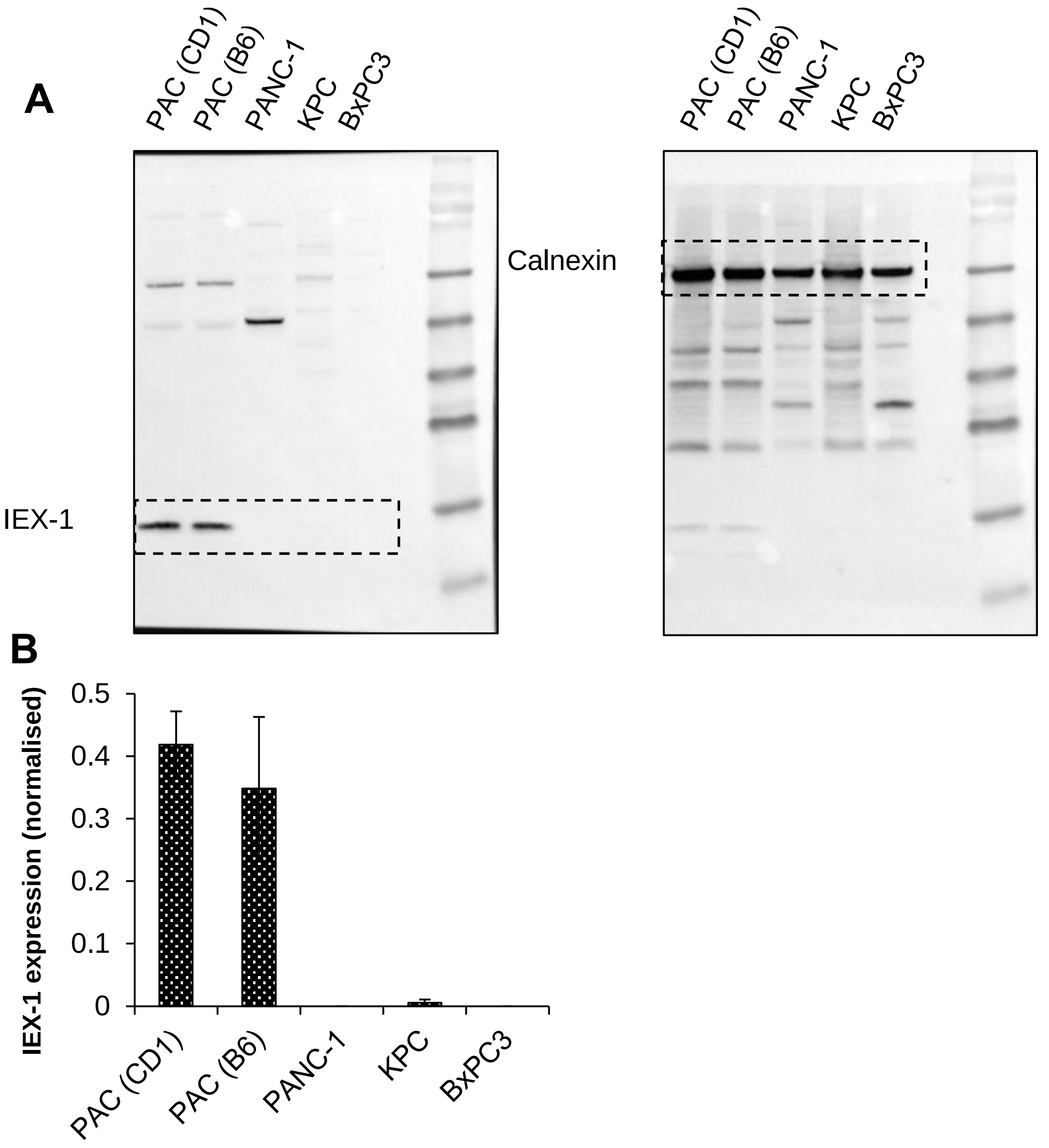

Supplementary Figure 6. Expression of IEX-1 in pancreatic acinar cells, and pancreatic cancer cell lines. (A) Full-length blots of IEX-1 in pancreatic acinar cells and pancreatic cancer cell lines. Cell lysates were firstly blotted for IEX-1 (left panel), and then re-blotted for calnexin (right panel) as a loading control. **(B)** Quantification of Western blot bands demonstrate that pancreatic cancer cell lines have significantly lower levels of IEX-1 than pancreatic acinar cells (PAC) from CD1 and C57BL/6 (B6) (n=3). The graph shows the intensities of IEX-1 bands normalised by the corresponding intensities of the calnexin bands.

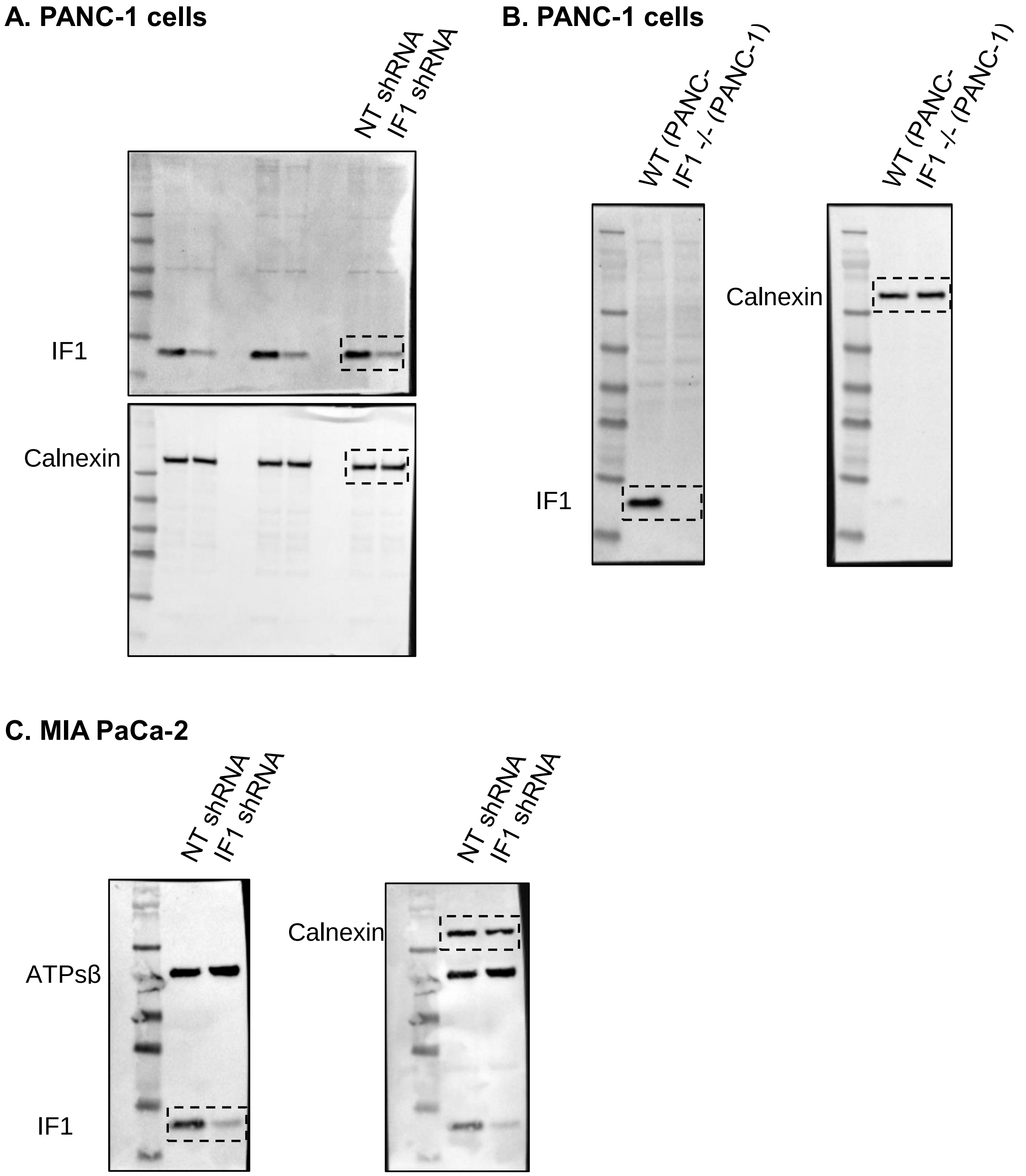

Supplementary Figure 7**. Full-length blots for IF1 in IF1 knockdown and knockout PDAC cells. (A)** Full-length blot of PANC-1 cell lysates corresponding to the Figure 5D in the main text; cell lysates were firstly blotted for IF1 (top panel) and then re-blotted for calnexin (bottom panel) as a loading control. **(B)** Full-length blots of PANC-1 cell lysates corresponding to the Figure 5D in the main text; cell lysates were firstly blotted for IF1 (left panel), and then re-blotted for calnexin (right panel) as a loading control. **(C)** Full-length blots of MIA PaCa-2 cell lysates corresponding to the Figure 5D in the main text; cell lysates were firstly blotted for IF1 (left panel) and then re-blotted for calnexin (right panel) as a loading control.

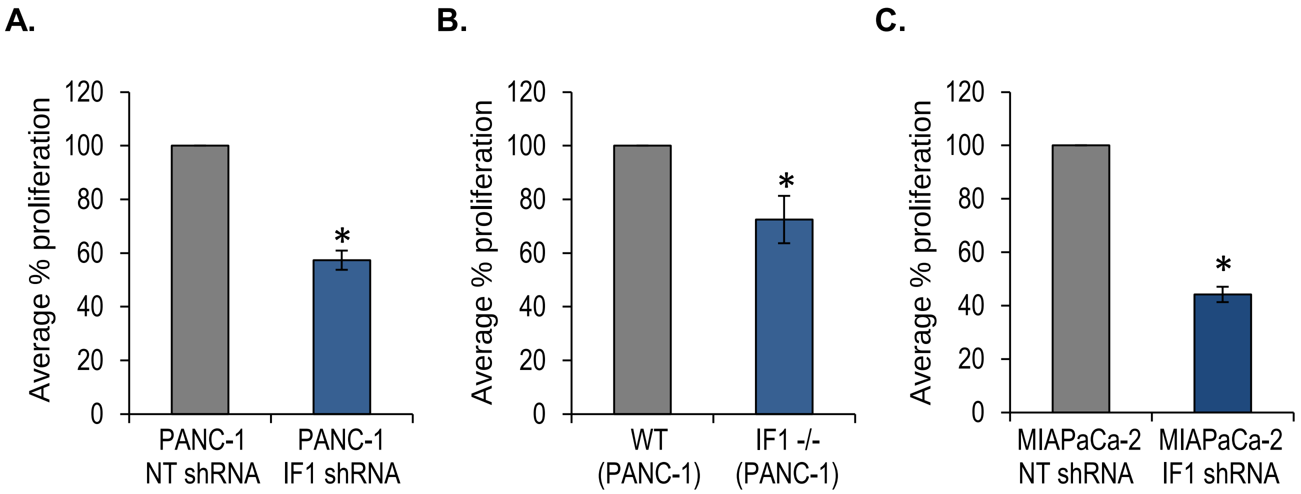

Supplementary Figure 8. The effect of IF1 knockdown and knockout on proliferation of PDAC cell lines. Percentage proliferation of PDAC cell lines was determined by taking 20 representative images of the cells on the day of seeding and 48hrs after seeding (A) The percentage proliferation of PANC-1 cells transfected with IF1 shRNA was normalised to that of cells transfected with NT shRNA (n=4) (B) The percentage proliferation of IF1-/- PANC-1 cells was normalised to that of WT PANC-1 cells for each individual experiment (n=4) (C) The percentage proliferation of MIA PaCa-2 cells transfected with IF1 shRNA was normalised to that of cells transfected with NT shRNA for each individual experiment (n=4).
